# Comparison between nasopharyngeal swab and nasal wash, using culture and PCR, in the detection of potential respiratory pathogens

**DOI:** 10.1186/1756-0500-4-122

**Published:** 2011-04-13

**Authors:** Jenna F Gritzfeld, Paul Roberts, Lorna Roche, Sherouk El Batrawy, Stephen B Gordon

**Affiliations:** 1 Respiratory Infection Group, Liverpool School of Tropical Medicine, Pembroke Place, Liverpool, UK; 2 NIHR Biomedical Research Centre, Directorate of Infection and Immunity, Royal Liverpool and Broadgreen University Hospitals NHS Trust, Liverpool, UK; 3 Royal Liverpool Hospital, Cheshire and Merseyside Comprehensive Local Research Network, Liverpool, UK

## Abstract

**Background:**

Nasopharyngeal carriage of potential pathogens is important as it is both the major source of transmission and the prerequisite of invasive disease. New methods for detecting carriage could improve comfort, accuracy and laboratory utility. The aims of this study were to compare the sensitivities of a nasopharyngeal swab (NPS) and a nasal wash (NW) in detecting potential respiratory pathogens in healthy adults using microbiological culture and PCR.

**Results:**

Healthy volunteers attended for nasal washing and brushing of the posterior nasopharynx. Conventional and real-time PCR were used to detect pneumococcus and meningococcus. Statistical differences between the two nasal sampling methods were determined using a nonparametric Mann-Whitney U test; differences between culture and PCR methods were determined using the McNemar test.

Nasal washing was more comfortable for volunteers than swabbing (n = 24). In detection by culture, the NW was significantly more likely to detect pathogens than the NPS (*p *< 0.00001). Overall, there was a low carriage rate of pathogens in this sample; no significant difference was seen in the detection of bacteria between culture and PCR methods.

**Conclusions:**

Nasal washing and PCR may provide effective alternatives to nasopharyngeal swabbing and classical microbiology, respectively.

## Background

Bacterial colonization of the nasopharynx occurs early in life, with *Streptococcus pneumoniae*, *Haemophilus influenzae*, and *Moraxella catarrhalis *causing sinusitis or otitis media in colonized children [1]. Nasopharyngeal carriage of potential pathogens is important as it is both the major source of transmission and the prerequisite of invasive disease. Sampling techniques involved in the detection of nasopharyngeal colonization are quite varied. In adults, nasopharyngeal aspirate (NPA) cultures have been shown to be positive more often for pneumococci than oropharyngeal swab (OPS) cultures, but culture results were best when both types of specimens were taken [2]. A similar study [3] found that nasopharyngeal methods (either swabs (NPS) or nasal washing (NW)) detected 89% of *S. pneumoniae *colonization as compared to 30% for OPS. There was no difference between the NPS and NW in detecting *S. pneumoniae*.

Detection of carriage is of great importance as it can be used in epidemiology studies, as an endpoint in vaccine trials, and in experimental human carriage projects, as has previously been done with *S. pneumoniae *[4]. Limitations of detection in conventional microbiology have led to the development of PCR-based detection systems. The development of PCR-based serotyping systems could aid in the surveillance of vaccine-targeted serotypes and help overcome difficulties associated with serological testing.

The present study aimed to compare the sensitivity of a nasopharyngeal swab to a nasal wash in healthy adults using microbiological culture and molecular techniques (PCR).

## Methods

### Recruitment

Healthy adults aged 18-60 were recruited to the study, which was given ethical approval by the National Health Service Research Ethics Committee (08/H1001/52) and was sponsored by the Royal Liverpool and Broadgreen University Hospitals Trust. Both a NPS and NW were used to determine if a volunteer was carrying a potential pathogen in the nasopharynx. Volunteers positive for carriage of potential pathogens were asked to return for serial sampling.

### Sampling

**Figure 1:**
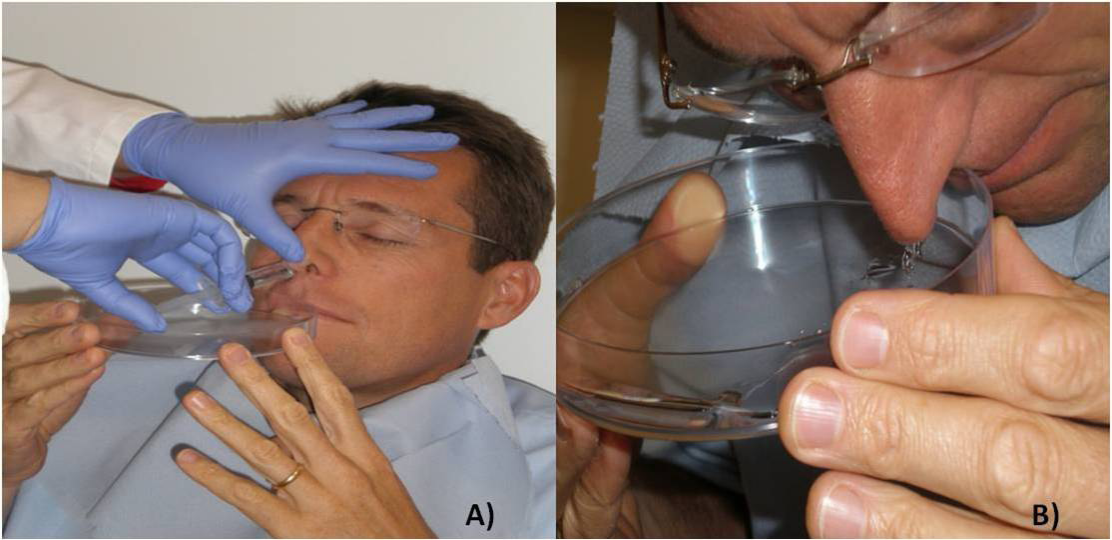
**Naclerio method of nasal washing**. A) A volunteer's head is tilted back 30° from the vertical and a syringe containing 5 ml of 0.9% sodium chloride is inserted into one of the nasal spaces. The volunteer briefly holds their breath while the 5 ml is expelled into the nasal space. B) Following a count of ten the volunteer leans forward and blows the fluid gently through the nares and into a Petri dish. The procedure is repeated with the other naris and the contents pooled.

Two consecutive specimens were taken from each volunteer; the NPS procedure was done first. The volunteer's head was tilted back slightly and a rayon-tipped flexible nichrome wire swab (Medical Wire, UK) was inserted into the naris until it reached the posterior nasopharynx. The rayon-tipped swab has been shown to be superior to calcium alginate and Dacron polyester swabs in the detection of pneumococcus [5]. The swab was rotated gently and then immediately placed into 1 ml of skim milk-tryptone-glucose-glycerine (STGG) medium for transport and processing. This procedure was repeated with the other naris. STGG medium was prepared as described previously [6]. The Naclerio method was used for the NW [7]. The volunteer's head was tilted back 30° from the vertical. A syringe filled with 5 ml of 0.9% sodium chloride was inserted into one of the nasal spaces and the volunteer was instructed to take a deep breath and hold it. The other nasal orifice (i.e., not containing the syringe) was held closed while the 5 ml of sodium chloride was expelled into the nasal space followed by a count of 10. The volunteer then leaned forward and blew the fluid gently through both nares onto a Petri dish (Figure 1). The procedure was repeated with the other naris and the contents were pooled into a universal container. The entire procedure was repeated twice more.

The Borg Scale of Discomfort, kindly provided by Dr. Frederick Hargreave, McMaster University, was used to determine the level of discomfort caused by the NPS and NW (0 = no trouble at all, 10 = maximum discomfort, similar to congestion from a 'cold').

### Classical microbiology

NPS specimens were vortexed in STGG medium and cultured on 7% horse blood agar and chocolate blood agar (Oxoid). NW specimens were centrifuged at high speed (1500 × *g *for seven minutes) and the pellet re-suspended in 1 ml STGG for storage; specimens were cultured on horse blood agar and chocolate blood agar. The remainder of the NPS and NW specimens were frozen at -80°C to be used for DNA extraction and PCR. All specimens were incubated for 24 hr at 37°C in 5% CO_2_.

Identification of *Staphylococcus aureus *was determined by catalase production and Pastorex Staph Plus latex test (Bio-Rad). *S. pneumoniae *was identified by colony morphology, alpha-haemolysis, optochin sensitivity, Gram stain, and bile solubility. *M. catarrhalis *was identified by colony morphology, Gram stain, a positive cytochrome oxidase test and hydrolysis of butyrate. Identification of *Neisseria meningitidis *was based on colony morphology, Gram stain, a positive cytochrome oxidase test and the API NH test strip (BioMérieux).

### Verification of pneumococcal recovery from NW samples

To determine if *S. pneumoniae *could be recovered from NW specimens containing normal nasopharyngeal flora, five NW samples were spiked with 0 μl, 0.2 μl, 1 μl, 10 μl, and 100 μl of *S. pneumoniae *23F stock culture. A viability count was performed on each spiked sample. The CFU of the starting culture was determined by a viability count and this was used to determine what concentration of *S. pneumoniae *23F had been added to each vial.

### PCR

DNA was extracted from the NPS and NW specimens using the QIAsymphony SP system and QIAsymphony Virus/Bacteria Midi Kit (QIAGEN Inc., UK) according to manufacturer's instructions. A total of 1200 μl was used in each reaction with an elution volume of 60 μl.

The conventional PCR assay for pneumococcus was taken from Pai *et al. *[8] using only the *cpsA *primer pair. The final reaction volume was 20 μl and the assay was performed using BioMix Red (Bioline Ltd.), according to the manufacturer's instructions.

The real-time PCR assay for meningococcus [9,10] was carried out in a final 25 μl reaction volume and was performed using TaqMan Universal Master Mix (Applied Biosystems) and 2.5 μl of sample DNA. DNA was amplified with the Corbett Rotor-Gene 6000 (QIAGEN) with the following cycling parameters: 50°C for 2 min, 95°C for 10 min, followed by 50 cycles of 15 s at 95°C and 1 min at 60°C. Amplification data were analyzed by instrument software (Rotor-Gene software series 1.7). Cycle threshold (C_t_) values ≤35 were considered positive; C_t _values between 36 and 40 were equivocal; and C_t _values >40 were called negative. DNA extractions of equivocal specimens were diluted 1:4 and 1:10 and re-tested in duplicate. If the resulting average C_t _values fell below 35, the specimen was considered positive. If the C_t _values remained >35, the specimen was called negative.

### Statistical analysis

Statistical analysis was performed using Microsoft Excel 2007, GraphPad Prism 4, and VassarStats (http://faculty.vassar.edu/lowry/propcorr.html, accessed 12-06-09). A distribution analysis was performed on data sets prior to examining statistical differences. A nonparametric Mann-Whitney U test was used to analyze the Borg Scale of Discomfort data. A McNemar test was used for the comparisons between NPS and NW. For all statistical comparisons a *p *value < 0.05 was defined as significant.

## Results

**Figure 2:**
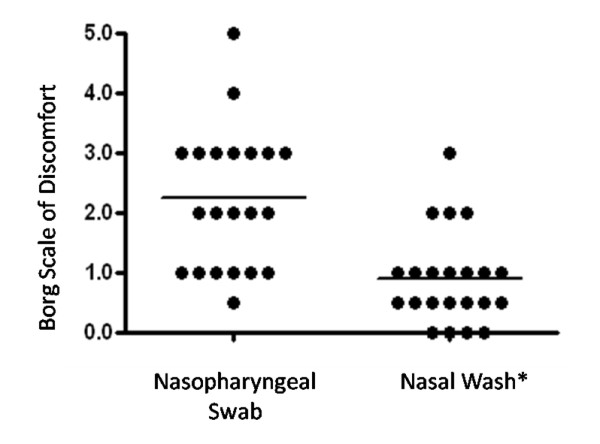
**Differences in the level of discomfort during NPS versus NW**. *p < 0.0001. NPS: 2.25 [1.74, 2.76]; NW: 0.89 [0.54, 1.23] (Mean [95% Confidence Interval]).

**Figure 3:**
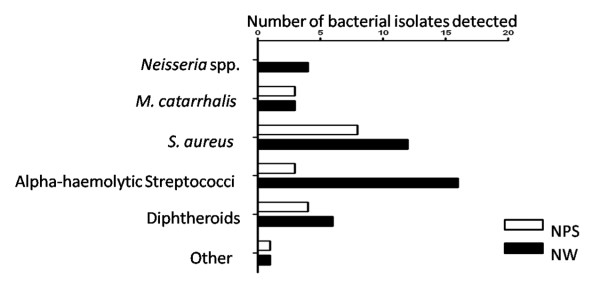
**Comparison of potential pathogens detected by NPS and NW**. (NPS, open bars; NW, filled bars).

Twenty-four volunteers were recruited to have a NPS and NW. Two volunteers chose not to have the NPS and three volunteers were sampled serially. Of the 22 people that had both the NPS and the NW, 91% preferred the NW while 9% found the NPS and the NW to be of equal discomfort (*p *< 0.0001) (Figure 2). NW was significantly more likely to detect potential pathogens than NPS (*p *< 0.00001). Of the bacteria detected in NPS specimens, 65% of isolates were non-*S. aureus *staphylococci. Of the potentially pathogenic organisms detected, 10% were *M. catarrhalis *and 26% were *S. aureus *(Figure 3). In the NW, 60% of isolates were non-*S. aureus *staphylococci. Of the potentially pathogenic organisms detected, 13% were *Neisseria *spp., 10% were *M. catarrhalis *and 38% were *S. aureus *(Figure 3). The lower limit for detecting *S. pneumoniae *23F in spiked NW specimens was 14 CFU/μl.

*S. pneumoniae *was not detected in any of the NPS or NW specimens but was detected in all four spiked NWs using the *cpsA *conventional PCR assay. The *sodC *real-time PCR assay detected *N. meningitidis *in NW more often than NPS but this result was not statistically significant (*p *< 0.25).

## Discussion

These results show that nasal washing is more comfortable than nasopharyngeal swabbing and is also more likely to detect potential pathogens. The Naclerio method of nasal washing has not been frequently used in the detection of bacteria; the most common method is a variation of instilling saline into the patient's nose and then suctioning it out through a tube, as was the method used by Lieberman *et al. *[3]. The Naclerio method would not be recommended in children because of the level of participant cooperation required but in adults it was a far easier method which proved successful in this study in both degree of comfort and pathogen detection. Others have tried several techniques (anterior nares, throat swabs) to reduce sampling discomfort but it is important in pneumococcal work to sample the posterior nasopharynx. Our findings are similar to those by Lieberman *et al. *[3] where NW was superior to NPS, especially in the detection of *H. influenzae *(*p *< 0.04).

The meningococcus real-time PCR is based on the detection of the *sodC *gene which encodes copper-zinc superoxide dismutase and is found in *N. meningitidis*, but not other *Neisseria *spp. [11,12]. The *sodC *assay is 99.6% sensitive and 100% specific for detecting *N. meningitidis *[13]. In this study, the only *N. meningitidis *culture positive sample was detected by real-time PCR, while *Neisseria polysaccharea*, *Neisseria cinerea *and *M. catarrhalis *were not detected using this assay. These results demonstrate that the *sodC *real-time PCR assay is sensitive and specific.

The combined NPS and NW results were examined but, due to the small sample size and the small number of volunteers found to be carrying *N. meningitidis*, the results were not significant. *N. meningitidis *was detected in only one culture sample and in two *sodC *PCR reactions. Only *N. meningitidis *could be analysed since *S. pneumoniae *was not detected by culture or conventional PCR.

There is currently an increased need for rapid detection of these potential pathogens, especially in relation to the recent H1N1 influenza A pandemic. In a group of U.S. patients with fatal pandemic influenza A, 29% showed evidence for a concurrent bacterial infection [14]. *S. pneumoniae *was strongly correlated with disease when NPS samples taken from patients with confirmed H1N1 infection were examined with MassTag PCR [15]. There is also an increased need for early recognition of bacterial pneumonia through easy-to-obtain samples and multiplex diagnostic methods. Current pneumococcal vaccine candidates are being designed to not only prevent pneumonia, but also prevent carriage and subsequently, transmission. Serial NW samples are a preferable sampling method to use in vaccine trials because of the increased detection capabilities and ease of administration as compared to NPS.

## Conclusions

NW was chosen by 91% of volunteers as being more comfortable than NPS and was more likely to detect pathogens using microbiological culture. Detection of *N. meningitidis *appeared to be better by real-time PCR on NWs than on NPSs but further testing remains to be done to confirm this trend.

## Competing interests

The authors declare that they have no competing interests.

## Authors' contributions

JFG undertook the study and data collection and produced the first draft of the article. PR gave laboratory support. LR and SEB collected the samples. SBG conceived the study. All authors read and approved the final manuscript.
